# Supplementary material for: The causal relationship between the human gut microbiota and pyogenic arthritis: a Mendelian randomization study
Source: Front Cell Infect Microbiol. 2024 Nov 26;14:1452480. doi: 10.3389/fcimb.2024.1452480 (PMC11629706; doi:10.3389/fcimb.2024.1452480)
Supplement: Supplementary file 1 [file DataSheet1.zip › mendelian test/ebi-a-GCST90027483.csv_forest.pdf]

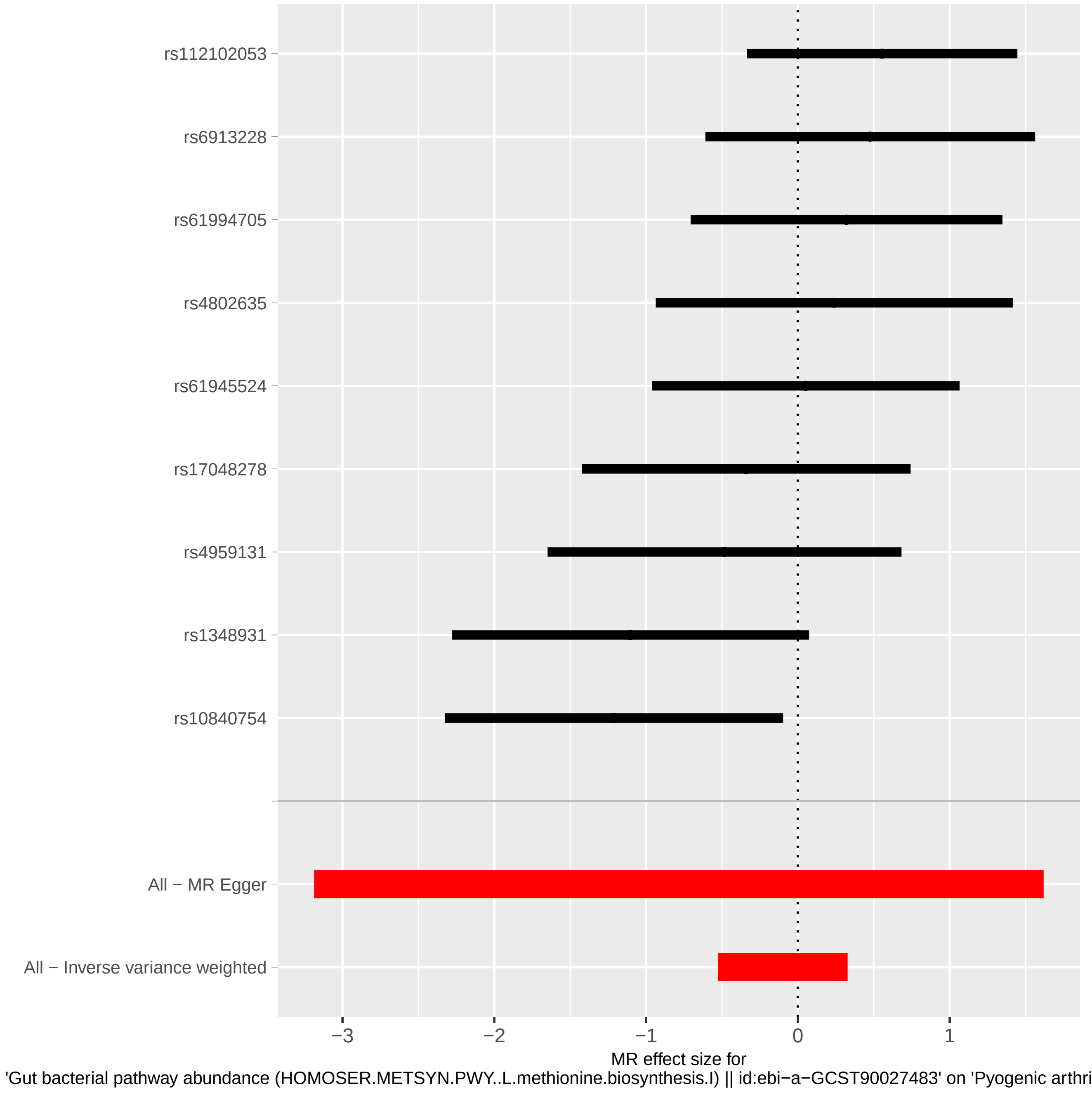
rs112102053
rs6913228
rs61994705
rs4802635
rs61945524
rs17048278
rs4959131
rs1348931
rs10840754
All − MR Egger
All − Inverse variance weighted
−3
−2
−1
0
1
MR effect size for
'Gut bacterial pathway abundance (HOMOSER.METSYN.PWY..L.methionine.biosynthesis.I) || id:ebi−a−GCST90027483' on 'Pyogenic arthri
